# The effect of a collective competence intervention on collective efficacy, psychological wellbeing, and social wellbeing: a quasi-experimental study of a sample of healthcare workers during the COVID-19 crisis

**DOI:** 10.3389/fpsyg.2024.1369251

**Published:** 2024-06-19

**Authors:** María Lourdes Campos, Pedro Bolgeri, Axel Bascur

**Affiliations:** Department of Psychology, Universidad de La Serena, La Serena, Chile

**Keywords:** collective efficacy, psychological wellbeing, social wellbeing, collective competence, intervention, COVID-19

## Abstract

**Background:**

The health crisis associated with COVID-19 led to a period of increased demand on the operational and social organization of healthcare centers, which often had a negative impact on the psychological and social wellbeing of healthcare workers. In order to tackle this issue, an intervention plan was designed to develop collective competences through various participatory strategies. This study sought to determine the effect of this intervention on the variables collective efficacy, psychological wellbeing, and social wellbeing in healthcare workers by performing a pretest and posttest comparison with a control group.

**Method:**

The variables were evaluated using a non-probability, purposive sample of 80 healthcare workers from three Family Healthcare Centers (CESFAM) located in the Coquimbo Region, Chile, within health crisis context. The intervention group was composed of voluntary participants, while the control group only completed the evaluations. The intervention consisted in 6 training workshops focused on improving collective management, group synergy, collaborative problem-solving, communicative strategies, and overall team care.

**Results:**

The analysis shows that the collective competence intervention had a positive effect on the collective efficacy, psychological wellbeing, and social wellbeing of the participating healthcare workers during the COVID-19 crisis. Only specific factors of these variables did not undergo a significant impact.

**Conclusion:**

The results of this study suggest that interventions aimed at improving collective organizational competences, apart from increasing collective efficacy, can have a positive impact on healthcare workers' psychological and social wellbeing in a context of occupational adversity.

## 1 Introduction

During the coronavirus pandemic crisis of 2019 (COVID-19), an increase in risk factors affecting the wellbeing of healthcare workers was observed. Several studies worldwide noted the presence of high levels of depression, anxiety, insomnia, emotional distress, and burnout, along with elevated rates of psychological distress and suicidal ideation among workers during this period (Barello and Palamenghi, 2020; Ruiz-Fernández et al., 2020; Sahin et al., 2020; Organización Panamericana de la Salud, 2022; Lee et al., 2023). Research shows that these symptoms have a negative effect on workers' ability to make adequate decisions while performing their tasks, potentially leading to negative consequences for their long-term physical and emotional wellbeing (Moyo et al., 2022).

In this context, several studies have reported a link between these symptoms and various organizational and occupational conditions present in healthcare centers (Mohanty et al., 2019; Mento et al., 2020). These factors include insufficient staff, increased workloads, longer work shifts, high performance expectations from users, and even instances of physical or verbal violence from users and peers.

In Chile, public healthcare workers serve around 78% of the national population (González and Castillo-Laborde, 2019). The Superintendency of Social Security, in evaluations of the working conditions of these professionals, detected a high level of psychosocial risk (Superintendencia de seguridad social [SUSESO], 2020). These risks mainly consist in imbalances between work and personal responsibilities, a lack of social support from their organizations, and a high level of psychological demand associated with daily work activities. These risk factors have been exacerbated by the arrival of COVID-19 due to increased workloads, long workdays, and concerns over carrying the infection to their own families (Organización Internacional del Trabajo, 2020; Alvarado et al., 2021).

Given this situation, it's relevant to identify factors and strategies related to the promotion of greater wellbeing among healthcare workers, bearing in mind that they must be tailored to the characteristics and standards of the organizational functioning of healthcare centers. In this regard, collective efficacy is defined as an indicator of working and organizational conditions, both positive and negative for the workers' activities. In work settings, it has been conceptualized as a team's or an organization's belief or assessment of their own ability to achieve relevant results and objectives. It depends on workers' deployment of interpersonal and collective competences, and on the organization's support, delivered through resources that facilitate the deployment of these capabilities, such as adequate time, materials, space, and suitable personnel (Campos et al., 2020b). This notion of collective efficacy as an extension of the efficacy theory and social action presented by Bandura (1997), which emphasizes the importance of individual trust in collaborative actions and social coordination, that is, mutual help, reciprocal stimulation, the expansion of one's scope of action, role complementarity, and inter-subject monitoring of the contributions and activities of each party (Roselli, 2011). Goddard et al. (2000) define four factors for this construct: (1) positive group competences, which include planning, coordination-cooperation, communication, follow-up and feedback, conflict solving, collaborative problem solving, and team adjustment; (2) negative group competences, which include factors that reduce perceived collective efficacy such as uncertainty about other's abilities and negative feedback; (3) positive analysis of elements with an influence on tasks, consisting in the appreciation of human, material, motivational, and structural resources that facilitate the completion of work tasks; and (4) negative analysis of elements with an influence on tasks, which, on the contrary, reveal a problematized view of the availability of these organizational and contextual resources.

Through this definition of collective efficacy, we first hypothesized (H_1_) that an intervention focused on developing group competences (or collective competences) in healthcare workers, would have a positive impact in their perceived group competences, and in their analysis of elements with influence on their tasks, compared to a control group. We didn't consider an intervention with a direct focus on the analysis of elements with an influence on tasks, since the availability of these resources depends on organizational conditions and dispositions that are outside of our intervention capacity. However, according with our theoretical background, it is still expected for these perceptions to be influenced by the development of collective competences, arguing that a better evaluation and understanding of group competencies, would lead to a better use and evaluation of these contextual resources, since both perceptions are related at the analytic-interpretative level of each individual (Goddard et al., 2000).

Several studies have found associations between collective efficacy and positive effects for people in diverse contexts. Researchers have observed that competences that facilitate collective work (e.g., better communication, collaborative problem solving) and the subsequent development of a shared consciousness and mental models, are linked to better performance, higher expectations and constant efforts to attain common goals (Sleegers and Daly, 2012; Goddard et al., 2017), while also increasing team commitment and members' willingness to stay in the organization (McLarnon and Woodley, 2021). This type of efficacy has also been linked to a higher level of self-efficacy of individual team members, more adaptive leaderships, more work satisfaction and motivation among workers (López and Gallegos, 2014; Versland and Erickson, 2017; Alavi and McCormick, 2018; Guidetti et al., 2018), and even more physical proximity and familiarity among group members (Patel et al., 2012). Based on this evidence, we proposed our second hypothesis (H_2_), stating that the healthcare workers who attended the collective competences development program, will show a significant improvement of their collective efficacy, and will also show improvements in their psychological wellbeing and social wellbeing scores, compared to a control group. We defined psychological wellbeing and social wellbeing as variables indicative of the overall wellbeing of healthcare workers, according to previous evidence of benefits that collective efficacy has at individual and interpersonal levels.

Psychological wellbeing is described by Ryff and Keyes (1995) as a type of wellbeing obtained through personal growth and the optimum development of one's individual capacities, which are essential for attaining a satisfactory life. One of its defining characteristics is its eudaimonic approach, which, unlike the hedonic or gratification-focused approach, entails the development of personal qualities that benefit the person's adjustment to the environment, healthy functioning, the attainment of vital challenges, and the continuous effort to achieve life goals (Blanco and Diaz, 2005). It represents the level of psychological development of each individual as they interact harmoniously and satisfactorily with their surroundings and daily activities (Jorquera and González, 2021). Ryff (2014) Multidimensional Model of Psychological wellbeing comprises six factors: (1) Self-Acceptance, which is the ability to feel good and have positive attitudes toward oneself; (2) Positive Relations with Others, which is the ability to preserve social relationships and have trustworthy friends; (3) Autonomy, which entails maintaining one's individuality in multiple contexts, having strong convictions, and being independent; (4) Environmental Mastery, which is the ability to generate or choose environments suitable for meeting one's needs and desires; (5) Purpose in Life, which is the ability to define objectives and goals that give meaning to one's life trajectory; and (6) Personal Growth, which consists in being persistent enough to develop one's potential and thus grow as a person while increasing one's individual skills and capabilities as much as possible. According to this definition of psychological wellbeing, and in relation to our previous hypothesis, we proposed our third hypothesis (H_3_), stating that a group intervened in collective competences, will show improvements in the six dimensions of psychological wellbeing, in comparison to a control group.

On the other hand, social wellbeing is defined by Keyes (1998) as the individuals' appraisal of their circumstances and functioning in society. As this author states, social wellbeing is also a multidimensional construct, which endures over time, and argues that people with more social wellbeing usually have feelings of belonging with solid social ties; trust both in themselves and others, accepting the positive and negative aspects of their lives; feel useful within a collective; have confidence in the future of society, being aware of its potential for growth and its benefits; and regard life—and the world they live in—as full of meanings and possible goals. As social interactions tend to happen differently in organizational contexts, we conceptualize social wellbeing through the structural adaptation validated by Campos et al. (2020a) for the workplace environment, constituted by three factors: (1) Social Belonging, which is the positive assessment of the degree to which workers feel attached to the organization, encouraging a feeling of usefulness and fidelity to it; (2) Social Interaction, defined as the positive assessment of the qualities of one's coworkers and the organization as a social system, considering the quality of the bonds and interpersonal relationships established; and (3) Social Comprehension, which is the positive assessment of the worker's understanding of the social and administrative functioning of the organization, which enables them to understand social relations, ties and events in the workplace. In sense of this definition of social wellbeing, we proposed our fourth and last hypothesis (H_4_), stating that a group intervened in their collective competences, will show an improvement in the three dimensions of social wellbeing, in comparison to a control group.

Based on these variables, the objective of this study is to determine how, within the context of the COVID-19 health crisis, an intervention aimed at developing collective competences have an impact in the collective efficacy, psychological wellbeing, and social wellbeing in a sample of healthcare workers. With this direction, we proposed the following research hypotheses:

- Hypothesis 1: Healthcare workers that attended the collective competences development program show a significant improvement in their positive collective competencies and has a better analysis of influential elements in tasks, compared to the control group.
- Hypothesis 2: Healthcare workers that attended the collective competences development program show a significant improvement of their collective efficacy, psychological wellbeing and social wellbeing scores, compared to the control group.
- Hypothesis 3: The intervention group shows a significant improvement in the dimensions of psychological wellbeing, self-acceptance, interpersonal relationships, autonomy, environmental mastery, purpose in life and personal growth, compared to the control group.
- Hypothesis 4: The intervention group shows a significant improvement in their social wellbeing, that is, greater social belonging, social interaction and social understanding, compared to the control group.

## 2 Materials and methods

### 2.1 Design

A quasi-experimental, pretest-posttest design was employed. Research variables were measured in an intervention group that participated in a training program aimed at developing collective competences and in a control group that took part in the evaluations, but not in the training program. The “quasi-experimental” denomination means that groups of participants maintained their natural composition instead of being randomly arranged (Hernández-Sampieri et al., 2014), since we needed to measure the variables on the organizational structure the subjects already maintained. For both groups, the variables were measured before and after the intervention.

To analyze the research variables, we implemented a repeated measures design, which makes it possible to evaluate the effect of an independent variable on a set of dependent variables. The dependent variables are collective efficacy, psychological wellbeing, and social wellbeing. The independent variables are those defined through the research design, such as the division between intervention and control group and the two evaluation phases (pre and post), as well as those external to the design, such as gender, age, and years of experience.

### 2.2 Participants

The participants were 80 healthcare workers from three public Family Health Centers (CESFAM) located in three municipalities of the Coquimbo Region, Chile. The subjects were selected non-probabilistically and purposively, as each group was composed of stable members from the same work team who needed to have been part of it for at least 6 months. The participating healthcare workers were 38.1 years old on average (SD = 10.8), ranging from 23 to 63 years of age, and had a mean of 13 years of work experience (SD = 10.6), ranging from 1 to 40 years.

The sample was grouped according to the subjects' participation in the intervention workshops, with 49 subjects being placed in the intervention group and 31 in the control group. Table 1 presents a detailed description of the characteristics of the groups and their composition.

**Table 1:** Descriptive analysis of the participants.

**Variable**	**Intervention group**		**Control group**		**Total**	
	***n***	**%**	***n***	**%**	***n***	**%**
**Age**						
20–29 years	10	20.4	5	16.1	15	18.75
30–39 years	22	44.9	12	38.7	34	42.5
40–49 years	9	18.4	10	32.3	19	23.75
50–59 years	5	10.2	4	12.9	9	11.25
60–69 years	3	6.1	0	0	3	3.75
**Gender**						
Male	15	30.6	10	32.3	25	31.25
Female	34	69.4	21	67.7	55	68.75
**Years of experience**						
1–9 years	19	38.8	11	35.5	30	37.5
10–19 years	19	38.8	12	38.7	31	38.75
20–29 years	7	14.3	8	25.8	15	18.75
30–39 years	3	6.1	0	0	3	3.75
40–49 years	1	2	0	0	1	1.25
**Occupation**						
Nursing	6	12.2	5	16.1	11	13,75
Speech-language pathology	2	4.1	2	6.5	4	5
Physical therapy	4	8.2	3	9.7	7	8.75
Obstetrics	3	6.1	2	6.5	5	6.25
Medicine	5	10.2	5	16.1	10	12.5
Nutrition	3	6.1	1	3.2	4	5
Dentistry	2	4.1	2	6.5	4	5
Psychology	3	6.1	2	6.5	5	6.25
Certified nurse technician	11	22.4	7	22.6	18	22.5
Occupational therapy	1	2	1	3.2	2	2.5
Others	9	18.4	1	3.2	10	12.5

### 2.3 Intervention design

The intervention process was informed by the “Competence-Based Psychosocial Development Model” (MIN-BAC), developed by Campos (2009) within the context of FONDEF Project DO5I10410. This model, based on social constructionism, seeks to highlight the dynamics and social experience transactions that make up the subject's reality and environment. It integrates the contributions of symbolic interactionism, including the interactions that people establish daily and through which they share subjective meanings of their own reality; furthermore, it incorporates Mead's view [1973, in Arnold-Garza (2014)] that the individual and their modes of action are constituted in social interactions. The model emphasizes the way in which actions take on symbolic meaning in a community and can ultimately become mediating conditions.

This model considers a series of strategic principles and procedures, applicable to various types of interventions. With respect to its principles, the model proposes that: (1) all social actors are important and enrich the process with their experience, (2) work is carried out in flexible stages, adapting the dynamic usage of each module as the process unfolds, (3) conditions are established and psychosocial strategies are applied in order to foster collaborative work by making a diagnosis, implementing development strategies, evaluating impact, and providing feedback on the intervention process with a view to attaining continuous improvements; and (4) the focus should be on the person (psychological factor) and their interactions (social factor), considering each subject as an active-reflective social constructionist who operates in context; in addition, active participation and interaction among social actors should be promoted.

**Figure 1:**
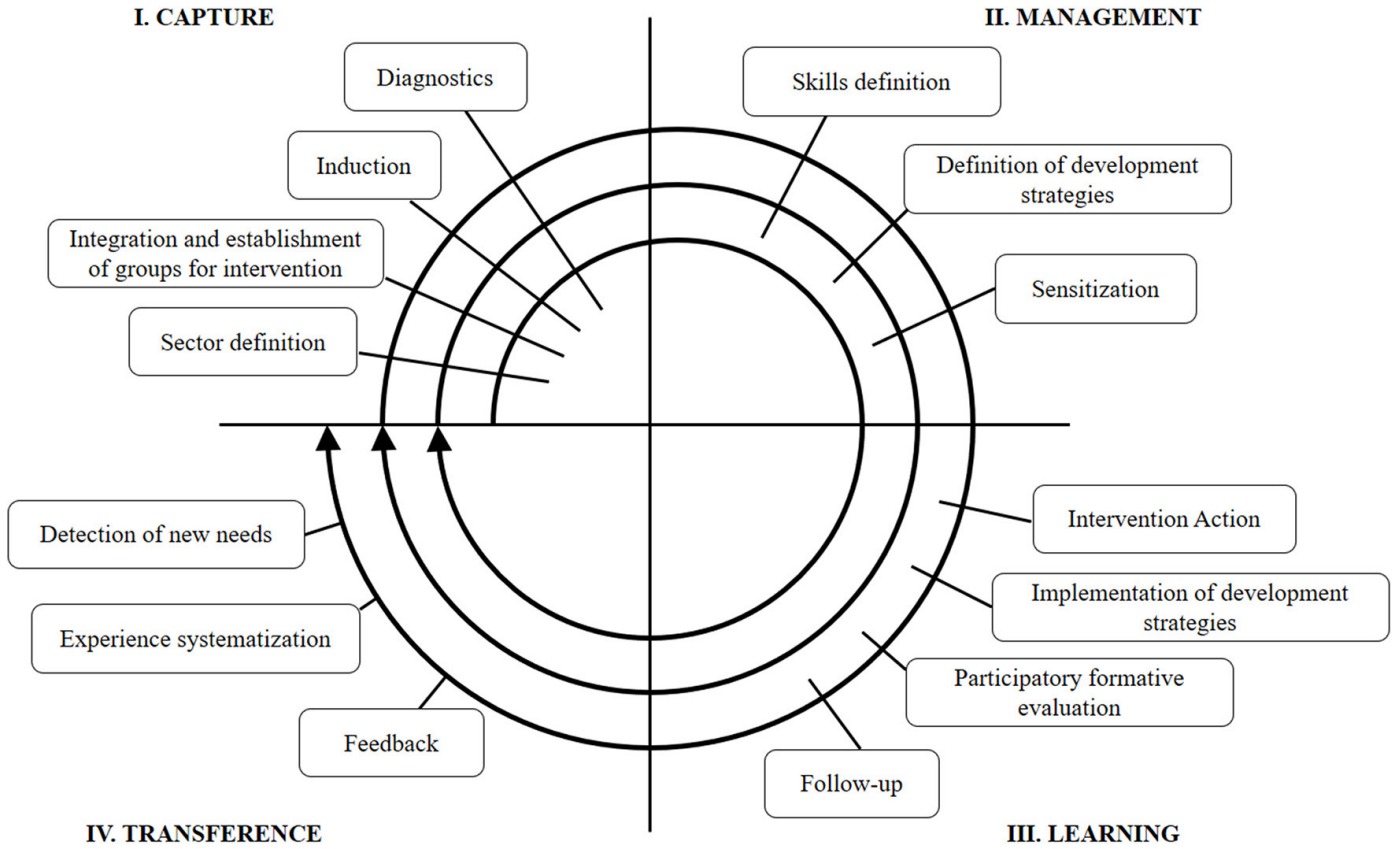
Competence-Based Development Model (MIN-BAC).

In addition, the intervention model comprises four successive intervention stages (capture, management, learning, and transference) and describes a set of steps to complete in each (see Figure 1).

In the capture stage, the induction was conducted through meetings with the authorities in charge of each center, who subsequently informed their workers of the initiative. Then, the diagnosis step was conducted by administering the instruments, equivalent to the pretest phase of the methodological design, applied around the first weeks of April 2022.

In the management stage, we defined a common intervention plan focused on the development of collective competences. The following specific intervention competences were defined: (1) collective management, establishing more democratic, horizontal, and integrative organizational dynamics; (2) collaborative problem solving, which means the development of an organizational consciousness, focused in sharing ways of understanding tasks, objectives, and other relevant aspects of the workplace; (3) communicative strategies that promote network-based functioning, involving strategies and benefits associated with collective and cooperative work, (4) the strengthening of group synergy, which comprises several measures aimed at facilitating coordination and the implementation of collective actions, and (5) team care, which promote team wellbeing through physical and emotional strategies (Ferrera and Gaete, 2012). These competences were spread across 6 sessions and covered through the explicit transference of definitions and specific knowledge related to each. This transference was complemented with integrative activities, whose aim is to make it easier for the participants to implement the knowledge acquired, activate certain behavior and experience domains, characterize their individual capabilities and the team's needs, and increase self-awareness and creative potential (Bolgeri, 2016).

The learning stage consisted in the execution of the workshops planned, which were conducted between April and September 2022. All six training sessions were carried out at the three healthcare centers (18 sessions in total), with an approximate duration of 4 h, and with a span of about 3 weeks between workshops, depending on the availability of the teams in the intervention group. In these workshops, following our schedule, we implemented the collective competence development and participative strategies. The intervention workshops applied focused, in principle, on fostering an environment of trust and openness among the participants. Then, the generation of proposals for transformative actions to address challenges in the field of health was promoted, and decision-making regarding the proposals through participatory processes. Subsequently, actions aimed at implementing the collectively prioritized strategies were coordinated. Lastly, the strengthening of interpersonal relationships and change processes within teams was encouraged, promoting teamwork and effective communication. The work method was characterized by active-participatory, experiential strategies contextualized in their real work experiences, and a focus on recreative and relaxing techniques that help with group integration and synergy. Then, 2 months after the completion of the workshops, around the last weeks of November 2022, we administered the follow-up posttests for each research variable.

Finally, during the transference stage, we systematized and analyzed the results obtained, a step that includes a feedback offered to the participant entities and social actors. In this process, we also consider the present study, as a way of sharing our findings and contributing to the development of disciplinary knowledge.

### 2.4 Ethical safeguards

For the present study, we established institutional collaboration agreements and complied with all the necessary ethical safeguards. As part of this effort, the Health Departments and the Confederation of Healthcare workers were asked to sign institutional informed consent documents. Workers who volunteered to participate in the study signed individual informed consent documents. This documentation explained how the participant's rights would be safeguarded, how their privacy would be protected, and how their data would be securely stored, certifying that they would only be used for research purposes.

### 2.5 Instruments

#### 2.5.1 Collective efficacy scale

Instrument adapted and validated by Campos et al. (2020b) for use in workplace settings, based on the scale developed by Goddard et al. (2000). It is composed of 19 Likert-format items, which are given scores ranging from 1 (strongly disagree) to 4 (strongly agree). It comprises four dimensions: group competence/positive (GC+), group competence/negative (GC–), task analysis/positive (TA+), and task analysis/negative (TA–). Its Cronbach's alpha has been calculated at 0.96.

#### 2.5.2 Ryff scale of psychological wellbeing

Instrument developed by Ryff (1989), adapted and translated into Spanish by Díaz et al. (2006). It is composed of 39 Likert-format items, which are given scores ranging from 1 (strongly disagree) to 6 (strongly agree). It comprises six dimensions: Self-Acceptance, Environmental Mastery, Positive Relationships with Others, Personal Growth, Autonomy, and Purpose in Life. Its Cronbach's alpha has been found to range from 0.68 to 0.83.

#### 2.5.3 Social wellbeing scale

Instrument validated by Campos et al. (2020a) for use in workplace settings; originally developed by Keyes (1998) and adapted and translated into Spanish by Blanco and Diaz (2005). It is composed of 13 Likert-format items, which are given scores ranging from 4 (strongly disagree) to 1 (strongly agree). It comprises three dimensions: Social Belonging, Social Interaction, and Social Comprehension. Cronbach's alpha for the overall scale has been calculated at 0.938.

### 2.6 Data analysis

Data processing and evaluation was performed using Jamovi 2.3.6. For the sociodemographic part of the data collection process, we employed descriptive statistical analysis, including each participant's age, gender, years of experience and occupation (Table 1).

We employed a data analysis approach consisting in the application of a General Linear Model, repeated measures ANOVA, along with partial eta squared, in order to examine the impact of the multiple experimental conditions on the indexes related to each dependent variable. This method makes it possible to compare the effects of the independent variables individually as well as the effect of their joint interaction on a specific dependent variable. We analyzed the dependent variables and each of their factors separately. This analysis yielded the descriptive statistics for the mean, standard error, 95% confidence intervals, *p*-values, and partial eta squared.

## 3 Results

**Figure 2:**
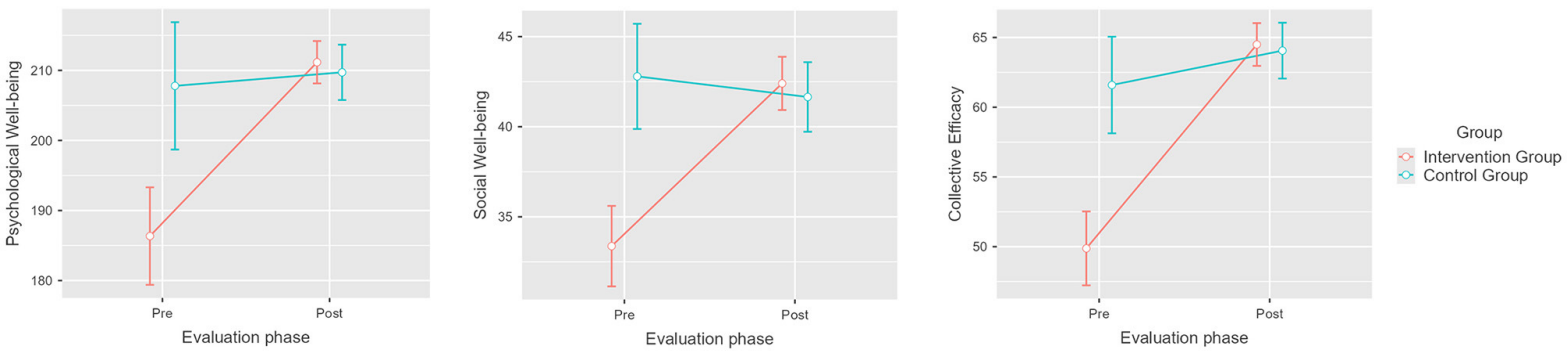
Pretest and post-test mean scores differences for psychological wellbeing, social wellbeing, and collective efficacy for the intervention and control groups. Error bars represent a 95% CI; significant differences between the intervention group and the control group. *p* < 0.001. Created with Jamovi 2.3.6.

Results show that the intervention had a significant effect at pretest and posttest for the three dependent variables [*F*_(1, 74)_ = 27.17, *p* < 0.001, η^2^*p* = 0.269]. Likewise, we found that the intervention had a significant effect on each individual variable, benefiting Collective Efficacy [*F*_(1, 74)_ = 21.94, *p* < 0.001, η^2^*p* = 0.229], Psychological wellbeing [*F*_(1, 74)_ = 12.90, *p* < 0.001, η^2^*p* = 0.148], and Social wellbeing [*F*_(1, 74)_ = 23.86, *p* < 0.001, η^2^*p* = 0.244]. In the intervention group, the Collective Efficacy mean increased by 15.2 points (M1 = 49.9, SE1 = 1.14; M2 = 65.1, SE2 = 0.71), the Psychological wellbeing mean increased by 24 points (M1 = 188, SE1 = 3.37; M2 = 212, SE2 = 1.40), and the Social wellbeing mean increased by 10 points (M1 = 32.9, SE1 = 1.02; M2 = 42.9; SE2 = 0.68; see Figure 2). The control group exhibited no significant differences in the dependent variables, with mean scores remaining similar at pretest and posttest evaluations: Collective Efficacy reached M1 = 61.2 (SE1 = 1.61) and M2 = 63.7 (SE2 = 0.87), Psychological wellbeing reached M1 = 205 (SE1 = 3.49) and M2 = 209 (SE2 = 1.4), and Social wellbeing reached M1 = 41.6 (SE1 = 1.21) and M2 = 41.5 (SE2 = 0.84). We found no significant effects associated with independent variables other than the effect of the intervention; therefore, we ruled out the influence of factors such as moment of evaluation by itself (*p* = 0.144), gender (*p* = 0.298), age (*p* = 0.442), and years of work experience (*p* = 0.844). These results verify our second hypothesis, as there was a significant improvement in collective efficacy, psychological wellbeing and social wellbeing scores for the intervened group (H_2_).

**Figure 3:**
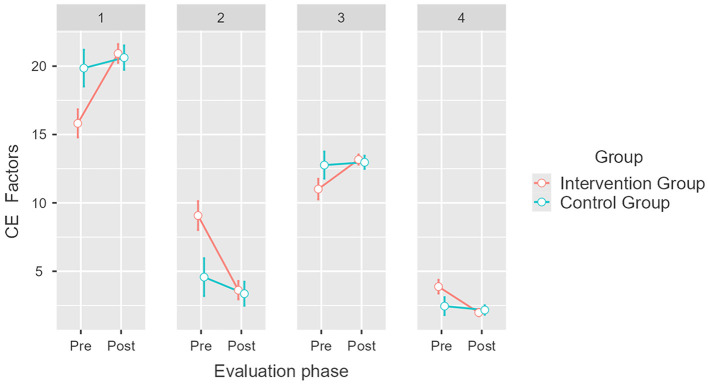
Pretest and post-test mean scores differences for Collective Efficacy (CE) factors for the intervention and control groups. Error bars represent a 95% CI; significant differences between the intervention group and the control group. *p* < 0.001. Created with Jamovi 2.3.6. 1 = group competence/positive (GC+), 2 = group competence/negative (GC–), 3 = task analysis/positive (TA+), 4 = task analysis/negative (TA–).

Regarding Collective Efficacy factors, we found significant differences between the means estimated for the intervention group depending on the phase of evaluation, which is indicative of the influence of the intervention [*F*_(3, 222)_ = 17.86, *p* < 0.001, η^2^*p* = 0.194]. For the intervention group, we found that the GC+ mean increased by 5.4 points, from M = 15.6 (SE = 0.46) to M = 21 (SE = 0.3); the GC– mean decreased by 5.67 points, from M = 8.98 (SE = 0.48) to M = 3.31 (SE = 0.31); the TA+ mean increased by 2.3 points, from M = 11 (SE = 0.37) to M = 13.3 (SE = 0.18); and the TA– mean decreased by 1.83 points, from M = 3.73 to M = 1.9 (SE = 0.12; see Figure 3). *Post hoc* analysis revealed no significant differences between the means of the control group (pbonferroni = 1). These results verify our first hypothesis (H_1_).

**Figure 4:**
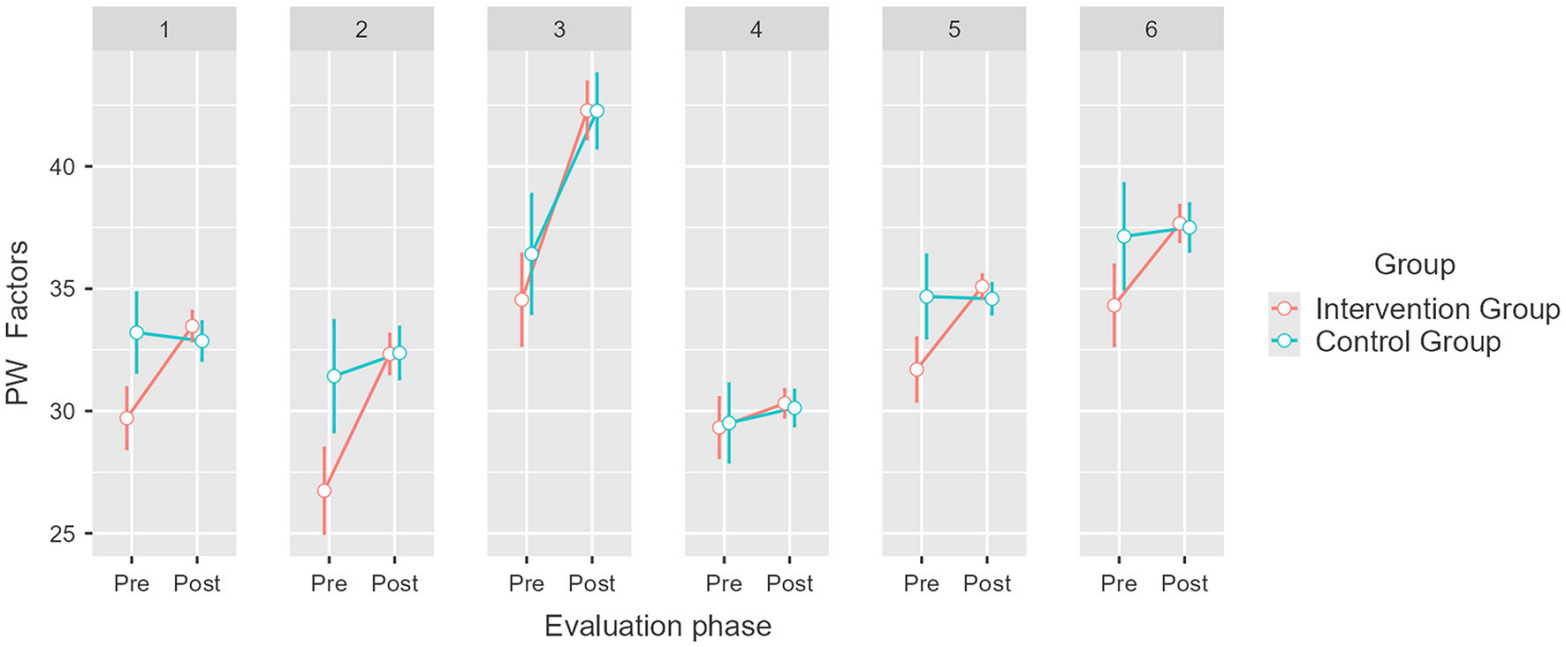
Pretest and post-test mean scores differences for Psychological wellbeing (PW) factors for the intervention and control groups. Error bars represent a 95% CI; significant differences between the intervention group and the control group. *p* = 0.008. Created with Jamovi 2.3.6. 1 = Self-Acceptance, 2 = Positive Relationships with Others, 3 = Autonomy, 4 = Environmental Mastery, 5 = Purpose in Life, 6 = Personal Growth.

With respect to PW factors, results also indicate a significant difference between the intervention group and the control group depending on the phase of evaluation, which is indicative of the influence of the intervention [*F*_(1, 74)_ = 7.47, *p* = 0.008, η^2^*p* = 0.092]. Thus, a significant effect is observed in the Self-Acceptance mean, which increased by 3.5 points (M1 = 29.9, SE1 = 0.58; M2 = 33.4, SE2 = 0.27); likewise, the Positive Relationships with Others mean rose by 5.4 points (M1 = 27.3, SE1 = 0.88; M2 = 32.7, SE2 = 0.37); the Autonomy mean increased by 7.1 points (M1 = 35, SE1 = 0.97; M2 = 42.1, SE2 = 0.54); and the Purpose in Life mean rose 3.4 points (M1 = 34.5, SE1 = 0.81; M1 = 37.9, SE2 = 0.35; see Figure 4). *Post hoc* analysis revealed no significant differences in Environmental Mastery or Personal Growth for the intervened group (pbonferroni = 1). *Post hoc* analysis revealed no significant differences in the control group' PW scores (pbonferroni = 1); except for the Autonomy factor, whose mean increased significantly by 6.6 points (M1 = 35.2, SE1 = 0.85; M2 = 41.8, SE2 = 0.67). These results partially confirm our third hypothesis (H_3_).

**Figure 5:**
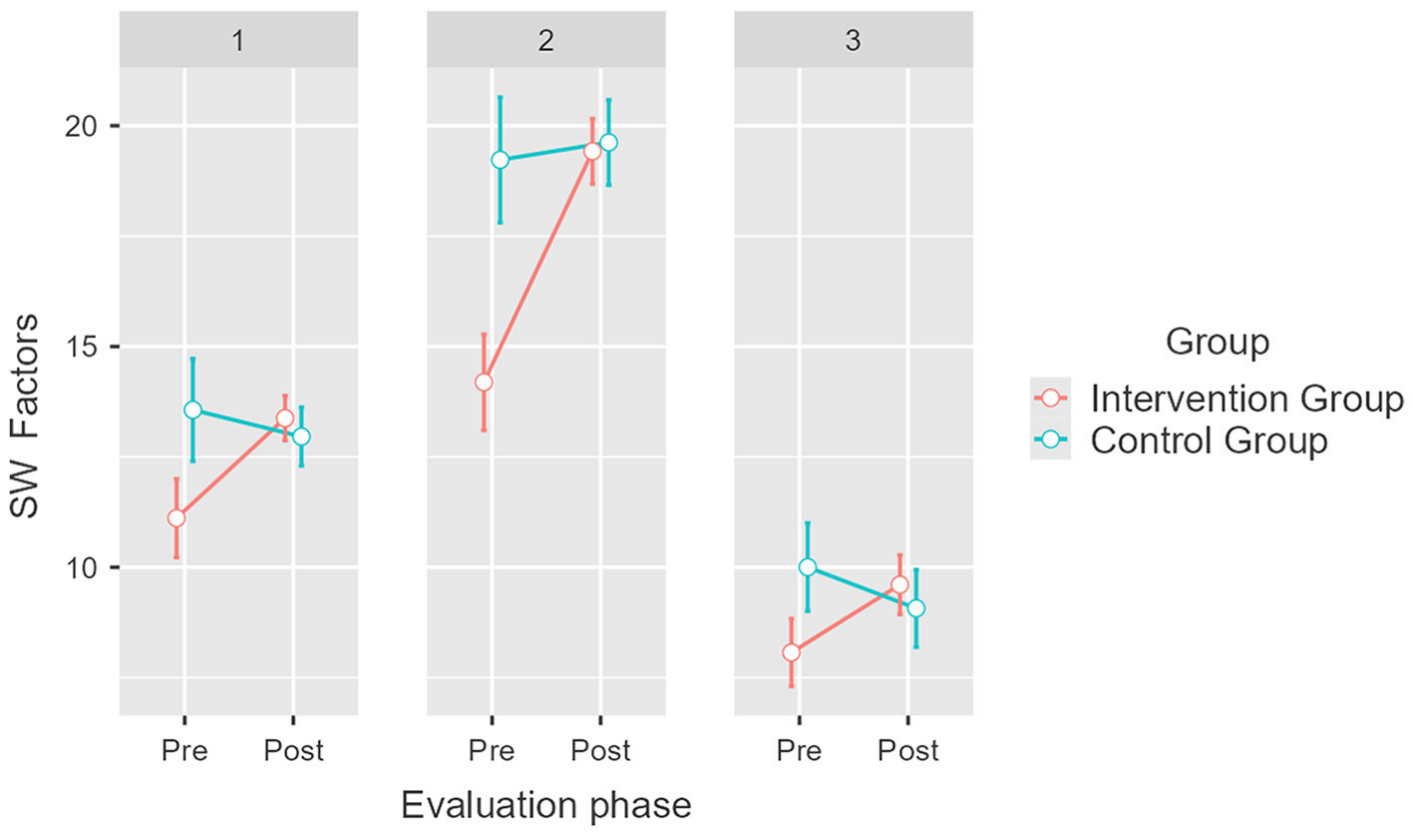
Pretest and post-test mean scores differences for Social wellbeing (PW) factors for the intervention and control groups. Error bars represent a 95% CI; significant differences between the intervention group and the control group. *p* = 0.005. Created with Jamovi 2.3.6. 1 = Social Belonging, 2 = Social Interaction, 3 = Social Comprehension.

With respect to SW factors, results also indicate a significant difference between the groups, considering the influence of the intervention [*F*_(2, 148)_ = 4.67, *p* = 0.011, η^2^*p* = 0.059]. In the intervention group, a significant difference was confirmed for the means of Social Belonging, which increased by 2.5 points, from M = 11.1 (SE = 0.44) to M =13.6 (SE = 0.24), and Social Interaction, which increased by 5.9 points, from M = 13.8 (SE = 0.49) to M = 19.7 (SE = 0.35; Figure 5). *Post hoc* analysis revealed that the Social Comprehension factor did not undergo significant changes in the intervention group [*t*_(74)_ = −311.7, pbonferroni = 0.172] and proved that there were no significant differences in the means obtained by the control group (pbonferroni = 1). These results partially confirm our fourth hypothesis (H_4_).

## 4 Discussion and conclusion

The goal of this study was to determine how, within the context of the COVID-19 health crisis, an intervention program aimed at developing collective competences influenced the variables Collective Efficacy (CE), Psychological wellbeing (PW), and Social wellbeing (SW) in a sample of healthcare workers. Our analysis of the resulting data allowed us to affirm that the intervention program had a positive and significant impact on all the variables studied compared to the control group, which did not take part in the intervention. Results underline the importance of generating collaborative competences that foster healthcare workers' psychological and social wellbeing, such as the ability to learn as part of a group and developing shared trust in the team's capacity to attain common goals (Stajkovic et al., 2009; McLarnon and Woodley, 2021).

The increase in Collective Efficacy derived from the intervention entails an improvement in the group's attitude and perceived capacity to achieve shared objectives, as well as a more positive appraisal of the elements that facilitate collective tasks (Campos et al., 2020b), along with a decrease in the problems perceived in these two dimensions. This means that the intervention plan presented attains its goal of developing collaborative competences, which suggests that these competences are also related to a change in the assessment of the resources available for collective work.

With respect to psychological wellbeing, our results suggest that this variable, while being a subject's individual measure of wellbeing, can be improved by the strengthening of collective competences and team functioning at an organizational level. In other words, actions that prompt a positive appraisal of the competences necessary for team performance can increase individual wellbeing, given that they help to increase self-acceptance and satisfaction with interpersonal relationships, promote a greater feeling of autonomy, and encourage a more clearly defined and relevant sense of purpose in life for each member of the work team (Díaz et al., 2006).

In the control group, the Autonomy dimension of Psychological wellbeing was the only one that exhibited a statistically significant change among measurements. This may be due to the fact that workers' opportunities to operate autonomously were curtailed by different restrictions implemented during the first stages of the pandemic (Lotta et al., 2020), but their sense of autonomy was almost immediately recovered, despite their subjective stress-levels not improving (Anicich et al., 2020).

With respect to social wellbeing, we also found an improvement related to the interventions' effect in developing healthcare workers' collective competences. This relationship, similar to our findings regarding psychological wellbeing, suggest that the reinforcement of collective competencies can help workers to feel more identified with, more useful, and more loyal to their organization and team members; while also promote a better assessment and quality of the bonds and interpersonal relationships established between them (Campos et al., 2020a).

These effects are consistent with the results of studies that associate workplace and organizational environment quality with the psychological wellbeing of healthcare workers (Brand et al., 2017; Ramaci et al., 2017), as the development of collective competences help team members to function more harmoniously in their work environments, enabling them to adapt, deal with challenges, and strive to achieve objectives that they deem to be relevant (Díaz et al., 2006; Moreta et al., 2017). Positive effects on social wellbeing are also consistent with studies that connect this variable with the fostering of collective efficacy, as the latter is associated with a better social climate and sense of community (Capone et al., 2017; Han et al., 2019).

The intervention was also expected to have a significant effect on all the factors that constitute each of the variables evaluated, but this was not matched by our results. With respect to psychological wellbeing, there were no significant changes in environmental mastery, that is, the ability to modify aspects of one's work environment to meet personal needs. This indicates that there is a separation between the promotion of competences derived from the intervention and the workers' effective capability to implement changes in their surroundings; which, in return, prompts some possible explanations: ranging from an organizational culture that may be too rigid for the healthcare centers included in the sample, which prevent changes in management, operations and perception of control in the personnel (Viinikainen et al., 2019); to that our intervention focus must be revised, as its' approach may not be sufficient regarding this capacity. Likewise, we found no significant differences in personal growth, which consists in the permanent motivation to improve one's skills. This may be due to the fact that, in the Psychological wellbeing instrument (Díaz et al., 2006), this is the factor with the lowest reliability index (α = 0.68), which reduces the capacity to differentiate the intervention group from the control group; but also because of the reasons explained before, at the organization and intervention level. As for social wellbeing, we found no significant differences in social comprehension, corresponding to the degree to which the worker understands the social and administrative functioning of the organization. This may have occurred because the intervention was planned and executed by actors external to the participating healthcare centers, which generated gaps in the integration of the informational tools specific to each organization' social context. Nevertheless, it is worth pointing out that these assertions must remain speculative because the specific causes of non-significant differences cannot be established, and also because the associations found could change if, for example, the tests were administered to larger samples.

The positive impact of the intervention on psychological and social wellbeing highlights the importance of developing collective competences, suggesting that similar group interventions could benefit healthcare systems, and also other organizational contexts. As suggested by Engestrom (2001), people's activity level and their capacity to perform tasks are expanded when they are able to face challenges and seek creative solutions through collaborative learning, a process that leads them to devise new ways to carry out tasks and achieve their goals. In the present study, the expansion of the participants' activity level gave rise to new ways of interacting, leading, and decision-making that benefited the functioning of the personnel and the organization, in addition to having positive implications for their wellbeing. This interrelationship is addressed by studies that link individual benefits, better at-workplace social interactions, and better organizational performance (Alshurideh et al., 2023; Park et al., 2023).

The type of intervention described in this article could be replicated in similar organizations that seek to improve collective efficacy and overall wellbeing, including their factors, and that expect to mitigate the mental health risks to which workers are exposed in public, service, or productive organizations (de Souza et al., 2018; Schneider et al., 2021), which is a highly valuable outcome in crisis contexts. Collective competences, as a factor that influences psychological and social wellbeing, can be actively integrated into the functioning of organizations as a protective factor benefiting workers' physical, mental, and social integrity. However, in order to attain these benefits, we emphasize the role of our intervention's design, which aims to maintain a continuous process with the participating teams, giving attention to their views, and working on the real challenges they encounter on their day-to-day tasks; opening a space for negotiation and proposal of practical solutions to be implemented in the work environment. This intervention scope is characterized for the promoting of employee wellbeing by offering relaxing and recreational activities, that are connected and build a progressive dialog between workshops, give attention to real working experiences, and compromise collective actions for intervention sustainability.

This study has several limitations that need to be considered. First, we must emphasize that our design is quasi-experimental, which implies the selection of the members of the intervention and the control group is not randomized. Therefore, the subjects who attended the workshops may have had specific capabilities or interests aligned with the topic or objectives of the program. This bias could explain the differences between the groups at the pretest stage, during which the intervention group had a lower mean than the control group across all evaluations. As this limitation hinders the generalizability of the results, we advise that randomized samples be employed in future research conducted under similar conditions. Likewise, given the limited sample size of our study, it is necessary to generate evidence using larger samples before attempting to generalize the effects presented in this article to the population.

Despite these limitations, the results obtained indicate that the intervention has the potential to contribute to the wellbeing of healthcare personnel, whose work environments subject them to risks of multiple severity levels. Importantly, the study also sheds light on the relationship between the competences that promote organizational efficacy and the psychological and social wellbeing of healthcare workers, considering that relations between these variables have received limited research interest. Furthermore, this study can inform future research capable of providing a clearer view of these workplace issues and defining the most suitable solutions. The results presented in this article delineate the importance of collective spaces and the impact of their characteristics on subjects and their interpersonal relationships, a perspective that can yield beneficial input not only in everyday situations but also during crises.

## Data availability statement

The raw data supporting the conclusions of this article will be made available by the authors, without undue reservation.

## Ethics statement

The studies involving humans were approved by Comité Ético Científico de la Unidad de Psicología de la Universidad de La Serena. The studies were conducted in accordance with the local legislation and institutional requirements. The participants provided their written informed consent to participate in this study.

## Author contributions

MC: Writing – original draft, Writing – review & editing. PB: Conceptualization, Project administration, Supervision, Validation, Writing – review & editing. AB: Investigation, Methodology, Validation, Writing – original draft, Writing – review & editing.
